# Dr. Edward Brown-Sequard’s Experimental and Clinical Researches, Epilepsy

**Published:** 1857-05

**Authors:** 


### SELECTIONS.

### *Dr. Edward Brown-Sequard's Experimental and Clinical Researches, applied to Physiology and Pathology.*

### EPILEPSY—(Continued).

SECTION XII. In many of the preceding parts of this paper I have strongly insisted on the influence of the aura epileptica, or of a peculiar kind of irritation of the peripheric nerves, as causes of epileptic fits. I must now show that I was right in this respect.

Herpin, in his important work which I have so often quoted (*loco cit.*, p. 421), tries to prove that the phenomena of the aura epileptica are nothing but the result of a cramp in one or in more muscles, and that this cramp is the first convulsion of the attack. The same view had already been proposed by Prichard, who says that the aura generally is "a convulsive tremor commencing in a limb" (A *Treatise on Diseases of the Nervous System*, Part first, 1822, *Note*, p. 88–89). Herpin has gone farther, and tried to prove that this is always the case. He thinks that the aura epileptica, or, in other words, the first cramp, depends upon a change in the nervous centres, and that the seat of the aura varies according to the place where the change begins in these centres. The cause of the attack, therefore, is in the cerebro-spinal axis, and the aura is only a manifestation, an effect, of this cause, and, in consequence, cannot be considered as a cause of the fit.

This theory implies that the so-called sympathetic epilepsy does not exist; it is a denial of the peripheric origin of epilepsy. I cannot understand such a denial, because I think there cannot be any doubt as regards the existence of the sympathetic epilepsy, when we take notice of the immense number of cases of this disease in which it has been produced by wounds or blows in various parts of the body, by neuromas, or other tumors, by dentition or decayed teeth, by foreign bodies, by worms, by calculi and other concretions, by diseases of the skin or of the trunks of nerves, &c. I will merely refer to the works of Portal (*loco cit.*, p. 155–185, p. 204–214), Esquirol (*loco cit.*, vol. i., p. 297–305), Delasiauve (*loco cit.*, pp. 217 and 253), and Romberg (*Nervenkrankheiten*, 3d ed., 1855, vol. i., part 2, pp. 689 and 700), where a great many such cases are reported.

When I treat hereafter of the nature and seat of epilepsy, I will try to show that almost always, if not always, there is in this disease an increased degree of the reflex excitability of the cerebro-spinal axis, and that epilepsy seems to consist mostly in this increased excitability. When a wound, or any of the known causes of the sympathetic epilepsy, produces this affection, it does so principally, if not only, by increasing this reflex excitability. I will show also, hereafter, that there are two distinct influences belonging to the various causes of the sympathetic epilepsy: by one, they produce the disease, or rather, the principal element of the disease, *i. e.*, an increase of the reflex excitability; by the other, they produce the fits. I refer, I repeat, to the writers I have just quoted, for facts proving that they may produce the disease, and I will now only try to show, in opposition to the theory of Dr. Herpin, that they often cause the fits. I will also try to show that many kinds of felt or unfelt irritation of the sensitive nerves of the skin, or of the muscles, have the same power.

A great many facts are opposed to the view that the aura results always from a cramp. In the first place, if this view were true, the sensation of the aura should always be felt where there are muscles, and not in those parts, such as the fingers, toes, skin, mammæ, testicles, ears, &c., where there are no muscles, and where, therefore, there cannot be any cramp. In taking notice only of cases reported by Herpin himself, in his learned historical account of the aura epileptica, we find that in a number of them the aura originated in the following parts: the little finger (two cases, one by Brassavola, the other by Hollier); the thumb (one case by Bouchet and Cazanvielh); a finger (one case by Faventinus); the big toe (four cases—two by Tulpius, one by Sylvius, and one by Portal); a cicatrix on the foot (one case by Puerari); all quoted by Herpin (*loc. cit.*, pp. 393, 394, 395, 398, 416 and 417). I might have given a much longer list by taking facts from other writers, ancient and modern. There are also many facts in opposition to the view of Herpin, in his own work, some of them observed by himself. There are cases in which there was a cramp, but, at the same time, a pain in parts where there was no cramp, and it is remarkable that the patients complained of this last pain only. So it was particularly in two cases observed by Herpin himself (Case xi., p. 70; and Case xix., p. 134).

If the view of Herpin were true, the sensations of the aura epileptica should be always the same, and always those of a cramp. Instead of such a thing, it is well known that these sensations vary extremely, and that they are described as a feeling of tickling, formication, burning, cold, &c. It would be easy to give a long list of cases in which these sensations have existed. Romberg, who admits two kinds of aura, a sensitive and a muscular one, says that the sensitive aura, in some of his patients, consisted of a feeling of formication in the extremities of the fingers and toes, and in others a tickling sensation around the mouth (*loco cit.*, p. 674).

Herpin says (p. 421–422), that he partially believes that epilepsy has been cured permanently or temporarily, and that the fits have been prevented, by stretching the limbs, frictions, ligatures, section of nerves, cauterizations, extirpation of parts, amputations, &c. These facts are certainly in direct opposition to his theory, and he feels much embarrassed about them. He tries, nevertheless, to show that there is no contradiction between his doctrine and these facts. His reasoning in this respect can prove only one thing, which is, that almost all the successful modes of treatment above enumerated are very powerful to diminish or prevent a cramp. But Herpin does not show how or why the prevention of a cramp cures epilepsy. Certainly it ought neither to cure the disease, or even to prevent the fit, as, according to the theory, the cause of the fit is in the nervous centres, and the aura, or first cramp or convulsion, is nothing but one of the effects of this cause. Of course, a cause is not destroyed, or rendered unable to act, because one out of many of its effects is annihilated. Herpin, very likely, has been aware of this inefficiency of his theory, as he tries to show—1st, that besides cauterization, in some cases powerful remedies have been employed; 2d, that he considers as doubtful some of the cases of cure by the extirpation of a tumor; 3d, that some operations have cured, for the same reason that fever and ague, typhoid fever, variola, &c., have.

I am surprised to find this last argument employed by Herpin, as there is nothing similar in the various operations performed for the cure of epilepsy, and these diseases. The alterations in the blood, and the changes in the nutrition of the nervous system which exist in these fevers may cure epilepsy, but in operations consisting in the application of a ligature round a limb, or in the section of a nerve, or in the extirpation of a tumor, there is nothing capable of altering materially the blood, and the nutrition of the nervous system. As to the other arguments of Herpin, they are valuable, but they apply only to a small number of cases.

It will be sufficient, I believe, to remind the reader of the cases of cure of epilepsy that I have given in a preceding section of this paper (see § IX). They prove peremptorily that the source of fits of epilepsy may be in the peripheric part of sensitive nerves. The fits were certainly due to an external cause of irritation in cases of epilepsy where they have been prevented by the following means:

1st. Application of a ligature around a limb or finger.
2d. Section of a nerve.
3d. Amputation of a limb, a finger, a toe, or the testicle.
4th. Extirpation of a tumor, a foreign body, or a tooth.
5th. Expulsion of worms, of calculi or other concretions.

Some other facts which I have mentioned in the beginning of § XI. show, in the most direct way, the possibility of the production of a fit by an irritation of the periphery of the sensitive nerves. Together with these facts, I might have spoken of a young man, observed by Zimmerman, and who had a fit of epilepsy every time he practised masturbation (Esquirol, *loco cit.*, p. 301). That an external irritation may cause fits is also proved, without any doubt, by the facts I have almost daily observed in animals for many years; facts described in the first part of these papers. It seems, even from what is observed in these animals, and from various circumstances observed in man, that when there is a cramp preceding a fit, the cramp is nothing but the first effect produced by the irritation of a sensitive nerve. Cramps in some of the muscles of the neck and face are sometimes the only effects of the excitation of the skin of the neck and face in my animals, and when a complete fit takes place, it is almost always preceded by the spasmodic contraction of these muscles. So that if we did not know that there had been an irritation of the skin, we might think that the first phenomenon was the cramp of these facial and cervical muscles. In cases of wounds of a nerve, two diseases may follow, epilepsy or tetanus, but in these two cases the first convulsive phenomenon is a cramp in the muscles in the neighborhood of the wound. Many facts of this kind have been collected by Swan (*A Treatise on the Diseases and Injuries of the Nerves*, new edition) and Pfluger (*Die sensorischen Functionen des Ruckenmarkes*, 1853). If the wound was not known to exist in a case of epilepsy of this kind, the local cramp would be considered as the first phenomenon of the attack, while it is only a secondary one. Now, if, as I have tried to show in § XI., there may be an unfelt irritation in the periphery of sensitive nerves, causing fits of epilepsy, it is possible that in cases where a cramp in one or a few muscles is the only thing felt by the patient, the cramp is not the first phenomenon, but results from the irritation of sensitive nerves in its neighborhood. In cases where there is both pain in a part without muscles, and cramps in the neighboring muscles, it may be that the cramps are the result of the irritation of sensitive nerves, causing this pain.

We do not deny that the first cramp in epilepsy may be due to some direct or primitive irritation of the nervous centres, but we do not know any cases where there was, in the beginning of the fits, a local cramp, resulting from a secondary irritation of the nervous centres, *i. e.*, produced by a reflex action, due to the excitation of sensitive nerves near the muscles attacked with cramp.

Romberg says that there are two kinds of aura epileptica; a sensitive and a muscular one. The sensitive consists of various sensations, the muscular consists in a cramp (*loco cit.*, p. 674). This distinction is more apparent than real. If there are cases where cramps are not reflex movements, depending upon the irritation of a sensitive nerve, and in which they result from the direct excitation of some parts of the nervous centres, such cases ought to be distinguished from those where a cramp is connected with an aura epileptica. Besides, when this connection exists, *i. e.*, when either a felt or an unfelt irritation of a sensitive nerve causes a local cramp by a reflex action, before it produces the other phenomena of a fit, the cramp is only apparently an aura.

In reality, there is only one kind of aura epileptica, if we leave to this word the meaning which it has had for centuries, *i. e.*, a local sensation preceding a fit. This sensation in some cases exists without any cramp in the neighborhood of its starting-point.

In cases where the irritation of a sensitive nerve causes a fit without being felt, there may exist a local cramp, but the name of aura cannot be given to this cramp, as it is only the first reflex manifestation of the preliminary irritation, the existence of which may be found out, as I have said, in § XI.—*Boston Medical and Surgical Journal.*

